# Facteurs associes a l'asthme sévère chez les patients asthmatiques suivis au Centre National Hospitalier de Pneumo-phtisiologie de Cotonou (Benin) en 2014

**DOI:** 10.11604/pamj.2015.22.11.5625

**Published:** 2015-09-07

**Authors:** Bella Adodo Hounkpe–Dos Santos, Akpa Raphaël Gbary, Alphonse Kpozehouen, Ferdinand Kassa

**Affiliations:** 1 Ministère de la Santé, Bénin; 2 Institut Régional de Santé Publique, Université d'Abomey-Calavi, Bénin; 3 Programme National de Lutte contre la Tuberculose, Bénin

**Keywords:** Asthme sévère, patients, facteurs associés, Bénin, severe asthma, patients, associated factors, Benin

## Abstract

**Introduction:**

la présente étude vise à déterminer la fréquence de l'asthme sévère chez les patients asthmatiques suivis au Centre National Hospitalier de Pneumo-Phtisiologie (CNHPP) de Cotonou et identifier les facteurs de risque qui lui sont associés

**Méthodes:**

l’étude transversale, descriptive et analytique a porté sur 213 patients asthmatiques de la file active 2013 du CNHPP. Les données ont été collectées par l'exploitation des dossiers et l'entretien individuel avec les patients. Elles ont été traitées et analysées à l'aide des logiciels EPIINFO7 et STATA11. Le test Chi^2^ de Pearson, la régression logistique uni variée et multi variée ont été utilisés au seuil de signification de 0,05

**Résultats:**

au total, 154 patients asthmatiques soit 72,7% ont répondu au questionnaire. Parmi eux 20,8% (IC_95%_:(14,67; 28,05)) souffraient d'asthme sévère. L’âge des patients s’étendait de 10 à 76 ans avec une médiane de 41 ans; 51,3% étaient de sexe féminin, 79,9% avaient des antécédents d'allergie, 61,7% ont commencé leur asthme après l’âge de 12 ans et seuls 11% ont consommé ou consommaient du tabac. Les facteurs associés à la survenue de l'asthme sévère étaient: l’âge de 46 à 55 ans (p = 0,04); les troisième et quatrième quintiles du bien-être économique (p = 0,01) et le début de l'asthme après l’âge de 12 ans (p < 0,001)

**Conclusion:**

l’étude a montré une fréquence élevée de l'asthme sévère au Bénin et permettra d'améliorer sa prise en charge au CNHPP.

## Introduction

L´asthme sévère est un asthme non contrôlé qui peut entraîner un risque d´exacerbations fréquentes graves, de mort, de réactions indésirables aux médicaments ou de morbidité chronique [1]. Les 40 dernières années, on a observé une augmentation de la prévalence de l'asthme sévère dans le monde et du fardeau économique qui lui est associé, en particulier chez les enfants [2, 3]. Au niveau mondial, on estime que l'asthme sévère touche entre 1 et 3% de la population générale chez les enfants et les adultes soit environ 5 à 10% voire 20% des patients asthmatiques [4, 5]. Les patients souffrant d´asthme le plus sévère consomment plus de médicaments, font plus appel aux médecins et sont plus souvent hospitalisés. L'asthme sévère génère donc un lourd poids économique pour les individus et leurs familles et aussi pour les pays [6, 7]. Il représente à lui seul 80% de dépenses de santé liées à l'asthme [8]. L'hospitalisation est responsable de plus de la moitié des coûts [3]. Plusieurs études récentes ont corrélé les dépenses liées à l'asthme, à la sévérité de la maladie et à la qualité du contrôle médical, montrant clairement qu'une partie importante des coûts est due à un contrôle sub-optimal de la maladie [3, 6].

Au Bénin, il y a peu de données sur l'asthme et rares sont les études épidémiologiques qui ont porté sur l'asthme sévère. L'asthme est l'une des pathologies prises en compte par le Programme National de Lutte contre les Maladies Non Transmissibles (PNLMNT). En outre, depuis 2009 il a été mis en place au Centre National Hospitalier de Pneumo Phtisiologie (CNHPP) une unité de prise en charge des malades asthmatiques en vue d'améliorer la prise en charge des patients ayant un asthme persistant en leur facilitant l'accès aux soins et un meilleur suivi. Malgré ces efforts, certains sujets continuent à être non contrôlés après une longue durée de suivi. Une étude réalisée au CNHPP en 2012 pour évaluer la prise en charge de l'asthme persistant a révélé que 83,2% des cas d'asthme n’étaient pas contrôlés après 6 mois à 1 an de suivi [9]. La sévérité de l'asthme est l'une des raisons essentielles qui rendent difficile le contrôle de la maladie. La présente étude avait pour objectifs de déterminer la fréquence de l'asthme sévère chez les patients asthmatiques suivis au Centre National Hospitalier de Pneumo-Phtisiologie (CNHPP) de Cotonou et d'identifier les facteurs de risque qui lui sont associés.

## Méthodes

***Cadre d’étude***: le CNHPP de Cotonou (Bénin) est la structure sanitaire nationale de prise en charge des malades tuberculeux et des autres affections pulmonaires. Il est situé à l'est de Cotonou à Akpakpa dans le quartier Abokicodji à environ 300 m du 2^ème^ pont de Cotonou en allant vers le cimetière d'Akpakpa. Le CNHPP est structuré en trois services medico-techniques qui sont le service de la prise en charge clinique, le service de radiologie et le laboratoire de référence des Mycobactéries. Il dispose également d'une pharmacie qui dispense aux malades les médicaments pour la prise en charge de l'asthme à des prix subventionnés.

***Type d’étude***: il s'agit d'une étude transversale descriptive et analytique menée d'avril à juillet 2014.

***Population d’étude***: les cibles de l’étude étaient les patients asthmatiques de la file active 2013 du CNHPP. Les sources de données étaient les dossiers médicaux des patients de la file active 203 du CNHPP et le registre de consultation de l'unité de prise en charge de l'asthme.

***Techniques d’échantillonnage***: le calcul de la taille de l’échantillon avec la formule de Schwartz n = z^2^_α_*p*q/i^2^, pour la proportion d'asthme sévère p = 10%, la précision i = 0,05 et le risque α = 0,05 avec Zα = 1,96 a donné n= 138. Ce nombre a été majoré de 10% pour prévoir les cas de non réponses et l'on a obtenu n =152. Cependant, les 213 dossiers de patients reçus pendant la période considérée ont été retenus. L'exhaustivité a été préférée pour accroître la puissance des tests.

***Variables à l’étude***: la variable dépendante a été l'asthme sévère. L'asthme sévère a été défini en tenant compte des orientations de l'OMS [1] pour les études épidémiologiques sur l'asthme sévère. Trois éléments ont été pris en compte pour cette définition: le niveau de contrôle (Tableau 1), la dose journalière de corticothérapie atteinte et l'observance du traitement. L'asthme était classé sévère si les 6 derniers mois, malgré une bonne observance du traitement, il a été partiellement ou non contrôlé malgré une dose moyennement ou très élevée de corticostéroïdes inhalée avec plus de 500µg de béclométhasone chez l'adulte et plus de 200µg chez l'enfant ou s'il a été contrôlé au prix d´une forte dose de corticothérapie inhalée avec plus de 1000µg de béclométhasone chez l'adulte et plus de 400µg chez l'enfant. Les données recueillies portaient sur les variables indépendantes suivantes: le sexe, l’âge, le niveau d'instruction, le milieu de résidence, le niveau de bien-être socio-économique, l’âge du début de l'asthme, les antécédents personnels d'allergie, les antécédents d'asthme ou d'atopie familial, l'exposition à la fumée de tabac ou à d'autres combustibles, les antécédents de réactions médicamenteuses, le milieu de résidence, l'exposition aux allergènes en milieu professionnel, les antécédents de maladie, le statut tabagique et le statut pondéral. Le niveau de bien-être économique du ménage dans lequel vit le patient a été déterminé selon le modèle de l'enquête démographique de santé du Bénin 2006 à partir de critères. Un score est affecté à chaque critère en fonction de la réponse du patient. Ce dernier est classé dans l'un des quintiles de bien être économique en fonction du score total obtenu [10]. Dans cette étude treize critères ont été retenues qui sont: appartenance de la maison où l'on vit, possession d'un terrain, sources d’énergie pour la cuisine, source d’éclairage, approvisionnement en eau, type de sol, type de toilette, toit de la maison, matériaux de construction, moyen de déplacement, source d'information dans la maison, moyen de communication et nombre de pièces utilisées par les membres de la famille pour dormir. Le statut pondéral des patients a été classé en maigre, normal, surpoids et obèse selon les critères de l'Organisation mondial de la santé [11].

**Tableau 1:** Niveaux de contrôle de l'asthme chez les sujets de plus de 5 ans

Caractéristiques	Contrôlé (1)	Partiellement contrôlé (2)	Non contrôlé (3)
	(1) toutes les caractéristiques	(2) et (3) au moins 1 des caractéristiques	
Symptômes journaliers	≤ 2jours/semaine mais pas plus d'1fois/jour	> 2 jr/semaine ou >1fois/ jour mais ≤2jours/semaine	Quotidien
Limitation de l'activité physique	Aucune	Oui (au moins une)	Très limitée
Symptômes et/ou réveils nocturnes	Aucun	≤2 nuits/ semaine	> 2 nuits/ semaine
Utilisation de β-2 mimétiques d'action rapide à la demande	≤ 2 jours/ semaine	>2 jours / semaine	Plusieurs fois dans la journée
Fonction Respiratoire (VEMS ou DEP)	≥80% (normale)	60-79% de la valeur prédite ou de la meilleure valeur personnelle	<60% de la valeur prédite ou de la meilleure valeur personnelle
Exacerbation ayant nécessité un corticostéroïde par voie orale ou systémique	0-1/an	2 / an	>2/ an

Source: Bousquet et al. Uniform definition of asthma severity, control, and exacerbations: document presented for the WHO Consultation on severe asthma. J Allergy Clin Immunol, 2010;126:926-938.

***Techniques et outils de collecte des données***: les techniques étaient basées sur l'utilisation de documents, l'enquête par questionnaire et l'observation. L'outil de collecte était constitué par une fiche de dépouillement des dossiers des patients, un questionnaire à l'endroit des patients, ainsi que du matériel de mesure tels qu'un pèse-personne, un mètre ruban et un débitmètre de pointe.

***Traitement et analyse des données***: les données ont été saisies à l'aide du logiciel EPIINFO version 7 et analysées par le logiciel STATA11.0. L´analyse a porté sur l´aspect descriptif et l´aspect analytique. Les caractéristiques de l’échantillon ont été décrites, les proportions estimées et les rapports de cotes ou odds ratio (OR) ont été calculés avec leur intervalle de confiance à 95% (IC95%) pour mesurer la force de l'association entre la survenue de l'asthme sévère chez les patients et les variables indépendantes. La comparaison des fréquences a été faite à l'aide du test de Chi2 de Pearson. Un modèle a été construit grâce à une analyse de régression logistique pas à pas descendante. Les variables ont été entrées dans le modèle multiple à partir de l'analyse uni-variée entre la variable dépendante et les variables indépendantes. L'adéquation du modèle final a été vérifiée avec le test d'Hosmer et Lemeshow [12]. Le seuil de signification pour tous les tests était de 0,05, mais les variables ont été introduites dans le modèle de régression multiple jusqu’à un seuil de 0,20.

## Résultats

Sur les 213 patients asthmatiques reçus en 2013 dont les dossiers ont été dépouillés, 154 ont répondu présents à la collecte de données et ont été interviewés, soit 72,3% de l'effectif attendu.

### Caractéristiques socio-démographiques des sujets interviewés

Ils étaient âgés entre 10 ans et 76 ans avec une distribution asymétrique de l’âge, la médiane étant de 41 ans avec un intervalle interquartile allant de 28,5 ans (Q1) à 54,5 ans (Q3). Cinquante trois pour cent étaient des femmes. Les sujets ayant atteint le niveau secondaire représentaient 32,5% des enquêtés. Plus d'un enquêté sur 3, se retrouvaient au niveau du troisième quintile du bien-être économique (Tableau 2).

**Tableau 2:** Répartition des patients asthmatiques suivis au CNHPP selon les différentes variables et le type d'asthme en analyse uni variée, Cotonou, Bénin, 2014

Variables	Modalités	Total (n = 154)	Asthme sévère (%)	OR	IC à 95%	p-value
**Sexe**	Féminin	79	21,5	1		
	Masculin	75	20,0	0,91	0,42 – 1,99	0,82
**Age**	=35 ans	58	12,1	1		-
	36-45 ans	33	9,1	0,73	0,17 – 3,03	0,66
	46-55 ans	28	42,9	5,46	1,84 – 16,22	0,00
	=56 ans	35	28,6	2,91	0,99 – 8,56	0,05
**Niveau d'instruction**	Primaire ou non scolarisé	59	27,1	1		
	Secondaire	50	16,0	0,51	0,20 – 1,32	0,17
	Supérieur	45	17,8	0,58	0,22 – 1,51	0,26
**Niveau de bien-être économique**	1^er^ et 2^ème^ quartile	88	13,6	1		
	3^ème^ et 4^ème^ quartile	66	30,3	2,75	1,23 – 6,15	0,01
**Age de début de l'asthme**	< = 12 ans	59	11,9	1		
	>12 ans	95	26,3	2,65	1,07 – 6,60	0,03
**Antécédents personnels d'allergie**	Non	31	29,0	1		
	Oui	123	18,7	0,56	0,23 – 1,38	0,20
**Antécédents d'asthme ou d'atopie familial**	Non	24	29,2	1		
	Oui	130	19,2	0,58	0,22 – 1,54	0,27
**Exposition à la fumée du tabac ou d'autres combustibles**	Non	117	18,8	1		
	Oui	37	27,0	1,60	0,68 – 3,78	0,28
**Antécédents de réactions médicamenteuses**	Non	133	21,1	1		
	Oui	21	19,1	0,88	0,27 – 2,83	0,83
**Milieu de résidence**	Urbain	91	23,1	1		
	Semi-urbain ou rural	63	17,5	0,70	0,31 – 1,59	0,40
**Exposition aux allergènes en milieu professionnel**	Non	39	15,4	1		
	Oui	115	22,6	1,61	0,61 – 4,25	0,34
**Antécédents de maladies**	Non	68	20,6	1		
	Oui	86	20,9	1,02	0,46 – 2,24	0,96
**Statut tabagique**	Non fumeur	144	20,8	1		
	Actuel ou ancien fumeur	10	20,0	1,05	0,21 – 5,22	0,95
**Statut pondéral**	Maigre ou normal	88	21,6	1		
	Surpoids ou obèse	66	19,7	0,89	0,40 – 1,96	0,77

### Autres caractéristiques des sujets interviewés

Les antécédents familiaux d'asthme ou d'atopie étaient fréquents chez les asthmatiques suivis au CNHPP (84,4%). Des 154 enquêtés, 79,9% ont déclaré avoir des antécédents personnels d'allergie et 38,3% ont commencé leur asthme avant l’âge de 12 ans. Seuls 24,0% des patients interviewés ne sont pas exposés dans leur milieu de vie à la fumée de tabac ou à d'autres combustibles. Près de la moitié d'entre eux (44,2%) souffrent d'au moins l'une des pathologies suivantes: le reflux gastro-œsophagien, les sinusites, la pneumonie, la dépression, l'anxiété et 13,6% ont un antécédent de réaction médicamenteuse connue à l'aspirine, à un anti-inflammatoire non stéroïdiens, à un antibiotique ou à un corticoïde. Un enquêté sur quatre (25,3%) a déclaré être exposé à un allergène comme la poussière, la fumée, l'odeur, les pollens et les animaux dans son milieu professionnel. Seuls 11% des patients interviewés ont consommé ou consommaient encore du tabac tandis que 42,9% sont en surpoids ou obèses (Tableau 2).

### Comparaison des non-répondants aux répondants

L’étude a enregistré 59 non-répondants, soit 27,7% des patients de la file active 2013. Les raisons de non-réponses étaient de plusieurs ordres. Trente patients n’étaient plus joignables à leur contact disponible au CNHPP, 4 étaient décédés, 15 étaient absents de Cotonou pendant la période de collecte des données pour l’étude et 10 patients n'ont pas répondu présents à l'invitation pour le questionnaire malgré les nombreux rappels. Cette proportion était élevée et nécessitait la comparaison des répondants avec les non répondants en fonction des variables indépendantes disponibles dans les dossiers qui étaient l’âge, le sexe, le milieu de résidence et le statut tabagique. Comme l'indique le Tableau 3, il n'y avait pas de différence significative entre le groupe des répondants et celui des non-répondants selon ces variables. Le groupe des répondants et celui des non-répondants étaient donc comparables.

**Tableau 3:** Répartition des patients asthmatiques selon qu'ils ont répondu ou non au questionnaire et selon les facteurs associés à l'asthme sévère au CNHPP de Cotonou, Bénin, 2014

Variables	Répondants (n = 154)		Non répondants (n = 59)		p-value
	Effectif	%	Effectif	%	
**Sexe**					0,87
Féminin	79	51,30	31	52,54	
Masculin	75	48,70	28	47,46	
**Age**					0,09
≤35 ans	58	37,66	26	44,07	
36-45 ans	33	21,43	4	6,78	
46-55 ans	28	18,18	13	22,03	
≥56 ans	35	22,73	16	27,12	
**Milieu de résidence**					0,517
Urbain	91	59,09	38	64,41	
Semi-urbain	56	36,36	17	28,81	
Rural	7	4,55	4	6,78	
**Statut tabagique**					0,09
Non fumeur	144	93,51	52	88,14	
Ancien fumeur	9	5,84	4	6,78	
Fumeur actuel	1	0,65	3	5,08	

### Fréquence de l'asthme sévère

Parmi les 154 enquêtés, 20,8% (IC95%: (14,67; 28,05)) souffraient d'asthme sévère.

### Facteurs liés à l'asthme sévère

Des quatorze variables étudiées, trois ont été significativement associées à la survenue de l'asthme sévère à l'analyse uni variée: l’âge et le niveau de bien-être économique et l’âge de survenue de la première crise. Ainsi, les sujets asthmatiques âgés de 46 à 55 ans avaient 5,46 fois significativement plus de risque de développer un asthme sévère que ceux âgés de 35 ans ou moins (p < 0,01). Les sujets âgés de 36 à 45 ans avaient moins de risque d'avoir un asthme sévère que ceux âgés de 35 ans et moins et les sujets de 56 ans et plus avaient 2,91 fois plus de risque que ceux âgés de 35 ans ou moins mais ces différences n’étaient pas significatives (respectivement p = 0,66 et p = 0,05). Les sujets appartenant aux troisième et quatrième quintiles du bien-être économique couraient 2,75 fois significativement plus de risque de développer un asthme sévère que ceux appartenant aux premier et deuxième quintiles (p < 0,01). Les sujets ayant débuté leur asthme après l’âge de douze ans avaient 2,65 fois (IC_95%_: (1,07; 6,6))significativement plus de risque de développer l'asthme sévère. Deux variables avaient des p-value < 0,20: les antécédents personnels d'allergie et le niveau d'instruction (Tableau 2). Ces cinq variables ont été introduites dans le modèle de régression multiple. (Tableau 4). En ajustant sur l’âge, le niveau de bien être économique, le niveau d'instruction et les antécédents personnels d'allergie, les sujets ayant débuté leur asthme après l’âge de douze ans ont 2,97 fois (IC_95%_: (1,03; 8,57)) significativement plus de risque de développer l'asthme sévère (p= 0,04). Lorsqu'on a ajusté sur le niveau de bien être économique, l’âge de début de l'asthme, le niveau d'instruction et les antécédents personnels d'allergie, les sujets asthmatiques âgés de 46 à 55 ans avaient 4,45 fois (IC_95%_: (1,4; 14,15)) significativement plus de risque de développer un asthme sévère que ceux âgés de 35 ans ou moins (p = 0,01). En prenant en compte l’âge des sujets, l’âge de début de l'asthme, le niveau d'instruction et les antécédents personnels d'allergie, les sujets appartenant aux troisième et quatrième quintiles du bien-être économique couraient 3,80 fois (IC_95%_: (1,53; 9,39)) significativement plus de risque de développer un asthme sévère que ceux appartenant aux premier et deuxième quintiles (p < 0,001).

**Tableau 4:** Répartition des patients asthmatiques suivis au CNHPP selon l’âge de début de l'asthme, l’âge, le niveau de bien-être économique et le type d'asthme en analyse multi variée, Cotonou, Bénin, 2014

Variables	Modalités	OR	IC à 95%	p-value
**Age de début de l'asthme**	<12 ans	1		
	>12 ans	2,97	1,03 – 8,56	0,04
**Age**	≤35 ans	1		
	36-45 ans	0,43	0,09 – 1,98	0,28
	46-55 ans	4,45	1,40 – 14,15	0,01
	≥56 ans	1,95	0,60 – 6,36	0,26
**Niveau de bien-être économique**	Le plus riche et le deuxième	1		
	Le moyen et le quatrième	3,80	1,53 – 9,39	0,00
**Niveau d'instruction**	Primaire ou non scolarisé	1		
	Secondaire	0,97	0,31 – 3,04	0,96
	Supérieur	1,24	0,37 – 4,18	0,72
**Antécédents personnels d'allergie**	Non	1		
	Oui	0,86	0,31 – 2,40	0,78

### Adéquation du modèle final

L'adéquation du modèle final vérifiée par le test de Hosmer et Lemeshow a donné un p= 0,71 confirmant que le modèle est adéquat.

## Discussion

### Comparaison des résultats avec ceux d'autres études

Les études sur la sévérité de la maladie asthmatique utilisent des variétés de définitions de l'asthme sévère qui diffèrent dans leur spécification en termes de fréquence et sévérité des symptômes et des exacerbations, de la fonction ventilatoire et de doses de corticostéroïdes [13–15]. Pour la discussion des résultats de cette étude, les comparaisons ont été faites avec les résultats des études explorant le contrôle de l'asthme et également ceux des études ayant utilisé diverses définitions de l'asthme sévère.

### De la fréquence de l'asthme sévère

La proportion de sujets asthmatiques souffrant d'asthme sévère dans cette étude est de 20,8%. Cette fréquence est élevée par rapport à la prévalence de 5 à 10% au niveau mondial [4, 5]. Elle est également élevée comparée aux 6% d'asthmatiques présentant des signes cliniques persistants sévères obtenu par l'Institut de Recherche et Documentation en Economie de la Santé (IRDES) en France [16]. La fréquence de l'asthme sévère au CNHPP est proche de la fréquence de 16% d'asthme persistant sévère trouvée par Aissa et al [17] en Tunisie dans une étude transversale, menée dans une consultation d'allergologie. Cette différence pourrait s'expliquer par le fait que l'IRDES a réalisé une enquête populationnelle tandis que la présente étude et celle d'Aissa et al ont été menées en milieu hospitalier. Le SARP [13] et l'ENFUMOSA [18] ont noté des fréquences plus élevées d'asthme sévère respectivement 46,6% et 50,8%.

### Des facteurs associés à l'asthme sévère

L’âge a été associé à l'asthme sévère. Cette association a été documentée par la plupart des études. Ainsi, dans l’étude SARP [13, 19], les sujets souffrant d'asthme sévère étaient plus âgés et souffraient de la maladie depuis plus longtemps 25 ±14 ans en moyenne contre 17 ±11 ans à 20 ±14 ans pour ceux souffrant respectivement d'asthme léger et d'asthme modéré. L’étude réalisée par l'IRDES [16] chez les asthmatiques de la population française a montré que les sujets âgés de 40 à 64 ans ont 1,47 fois significativement plus de risque d'avoir un asthme non contrôlé que ceux âgés de moins de 40 ans. Godard et al [20], en France métropolitaine, ont trouvé que le pourcentage de patients avec un contrôle inacceptable de leur asthme augmentait significativement avec l’âge. Toutefois, parmi les sujets souffrant d'asthme sévère, ceux faisant 3 exacerbations ou plus par an étaient significativement les plus jeunes [21]. Contrairement à nos résultats, Yildiz [22], dans une étude réalisée en Turquie pour évaluer les profils de patients asthmatiques et de leurs dispositifs de traitement de l´inhalateur en matière de contrôle de l´asthme n'a pas trouvé que l’âge était lié au contrôle de l'asthme. Il en est de même pour l'ENFUMOSA [18, 19] qui n'a pas trouvé de différence liée à l’âge entre les sujets présentant un asthme sévère et ceux ayant un asthme léger ou modéré. Le sexe n’était pas associé à l'asthme sévère dans notre étude. Notre résultat rejoint celui de Varraso et al [23], dans l´étude épidémiologique sur la génétique et l´environnement de l´asthme (EGEA), qui ont noté que la sévérité de l´asthme n´était pas liée au sexe lorsque l´on considère le score clinique, les stéroïdes inhalés, ou les hospitalisations au cours de la vie. Le SARP [13, 19] n'a également pas trouvé le sexe associé à l´asthme sévère entre les sujets souffrant d´asthme sévère et les sujets atteints d´asthme modéré ayant une fonction pulmonaire similaire. Par contre, Godard et al [20], à l'analyse bi variée comme à l'analyse multi variée, ont trouvé que la probabilité d'avoir un contrôle optimal de l'asthme était significativement associée au sexe.

Le niveau de bien-être économique a été significativement associé à l'asthme sévère dans notre étude. Blanc et al [24], ont noté que les asthmatiques dont les ménages proviennent de milieu de faible niveau socio-économique avaient un score de sévérité de l'asthme significativement plus élevé. Pour Eisner et al [25] les sujets ayant les plus faibles revenus ont été significativement plus à risque d'hospitalisations pour asthme durant les 18 mois de suivi dans leur étude. Cette disparité entre les différentes classes socioéconomiques pourrait résulter d'expositions plus importantes dans les quartiers moins favorisés à certains facteurs de risque psychosociaux et environnementaux pouvant induire ou exacerber l'asthme. Le pouvoir d'achat plus faible et une observance moindre du traitement dans les classes moins favorisées pourraient également rendre compte de ce résultat. Il n'y avait pas d'association significative entre le niveau d'instruction et l'asthme sévère dans cette étude. Yildiz [18] et Blanc et al [24], n'ont pas mis en évidence de lien entre le niveau d'instruction et contrôle de l'asthme ou le score de sévérité de l'asthme. Cette relation a cependant été retrouvée par l'IRDES [16]. Dans cette étude, les sujets les moins instruits avaient plus de risque d'avoir un asthme non contrôlé que les sujets les plus instruits. Eisner et al [25] ont observé dans une étude de cohorte réalisée en Californie du Nord que plus le nombre d'années d'instruction augmente, plus le risque d'hospitalisation pour asthme diminue. Cette divergence de résultats peut s'expliquer par les spécificités sociogéographiques locales.

Les antécédents d'asthme ou d'atopie familial et les antécédents personnels d'allergie n'ont pas été identifiés comme facteurs de risque de l'asthme sévère. Le SARP [13, 19], par contre, a trouvé que l'atopie confirmée par le test cutané était significativement moins fréquente chez les sujets souffrant d'asthme sévère à l'analyse uni-variée qu’à l'analyse multi variée. Cette association a été également retrouvée par l'ENFUMOSA [18, 19]. Dans une étude transversale réalisée par Amelink et al aux Pays-Bas [26] chez les sujets ayant développé leur première crise d'asthme à l’âge adulte, l'absence d'atopie a été l'un des facteurs associés à la sévérité de l'asthme à l'analyse univariée. Pour Ten Brinke et al [21], l'antécédent familial d'asthme a été significativement associé aux exacerbations fréquentes parmi les sujets souffrant d'asthme sévère. Les résultats obtenus dans la présente étude pourraient s'expliquer par le fait que la notion d'atopie et d'allergie a été recherchée par l'interrogatoire et non par des tests cutanés. Les sujets ayant débuté leur asthme après l’âge de 12 ans ont été plus à risque d'avoir un asthme sévère. Yildiz [22] n'avait pas noté une liaison significative entre l’âge de début de l'asthme et le niveau de contrôle. Le SARP [13] également n'a pas trouvé de différence entre les groupes selon l’âge de début de l'asthme, toutefois la survenue de l'asthme après l’âge de 12 ans était significativement associée à la réduction de la fonction pulmonaire et aux infections sinusopulmonaires chez les sujets souffrant d'asthme sévère. Selon Pascual et Peters [27], dans une revue d'articles, l'association de l’âge de début de l'asthme à la sévérité de la maladie reste discutable en raison des données contradictoires retrouvées par les différents auteurs.

Dans la présente étude, il n'y a pas eu de relation significative entre le tabagisme et l'asthme sévère. La faible proportion de sujets tabagiques actuels comme anciens pourrait expliquer ce résultat car, le tabagisme actuel comme passé a été retrouvé très souvent lié à l'asthme sévère et au non contrôle de l'asthme dans plusieurs études [14, 20, 22, 28]. Toutefois, ce résultat concorde avec ceux du SARP [13] et de l'IRDES [16] qui n'ont pas retrouvé d'association avec le tabagisme. Le surpoids et l'obésité n’étaient pas significativement associés à l'asthme sévère. Ce résultat concorde avec celui du SARP [19] aux Etats-Unis qui n'a pas trouvé l´obésité associée. Les études ENFUMOSA [19] et EGEA [23] ont, quant à elles, retrouvé cette association de l'obésité chez les sujets de sexe féminin. Aux Etats-Unis, une enquête nationale sur l'asthme [29] ayant inclus 3095 sujets adultes a observé que les sujets obèses étaient significativement plus susceptibles d’être classés comme asthme persistant sévère. L'IRDES [16] a noté que les sujets en surpoids et ceux obèses avaient significativement plus de risque d'avoir leur asthme non contrôlé que ceux ayant un poids normal ou étant maigres. Pour Godard et al [20], la probabilité d'avoir un contrôle optimal de l'asthme était significativement plus élevée chez les patients ayant un indice de masse corporelle normal par rapport aux patients obèses. Ces résultats contradictoires en relation avec l'indice de masse corporelle devraient faire l'objet d’études plus approfondies.

## Conclusion

La présente étude a permis de connaître la fréquence de l'asthme sévère parmi les asthmatiques suivis au CNHPP en se basant sur les nouvelles orientations de l'OMS pour les études épidémiologique sur la sévérité de l'asthme. Cette fréquence a été de 20,78%. Les facteurs associés à l'asthme sévère ont été également déterminées dans cette population d’étude. Trois facteurs ont été associés à l'asthme sévère. Il s'agit de l’âge élevé et du faible niveau de bien être économique et de l’âge de début de l'asthme supérieur à 12 ans. L'asthme sévère est un problème de santé publique qui n'est pas souvent pris en compte dans les priorités. Il est important de prendre les mesures en termes d'organisation de services au niveau du CNHPP et de communication pour un changement de comportement auprès des patients pour améliorer sa prise en charge. Par ailleurs, ces résultats peuvent servir à la réalisation d’études approfondies sur l'asthme sévère.
